# When a Text Is Translated Does the Complexity of Its Vocabulary Change? Translations and Target Readerships

**DOI:** 10.1371/journal.pone.0110213

**Published:** 2014-10-29

**Authors:** Hênio Henrique Aragão Rêgo, Lidia A. Braunstein, Gregorio D′Agostino, H. Eugene Stanley, Sasuke Miyazima

**Affiliations:** 1 Departamento de Física, Instituto Federal de Educação, Ciência e Tecnologia do Maranhão - IFMA, São Luís, Brazil; 2 Center for Polymer Studies, Boston University, Boston, Massachusetts, United States of America; 3 Departamento de Física, Facultad de Ciencias Exactas y Naturales, Instituto de Investigaciones Físicas de Mar del Plata (IFIMAR), Universidad Nacional de Mar del Plata-CONICET, Mar del Plata, Argentina; 4 ENEA – CR “Casaccia,” Roma, Italy; 5 Department of Natural Sciences, Chubu University, Kasugai, Aichi, Japan; University of Maribor, Slovenia

## Abstract

In linguistic studies, the academic level of the vocabulary in a text can be described in terms of statistical physics by using a “temperature” concept related to the text's word-frequency distribution. We propose a “comparative thermo-linguistic” technique to analyze the vocabulary of a text to determine its academic level and its target readership in any given language. We apply this technique to a large number of books by several authors and examine how the vocabulary of a text changes when it is translated from one language to another. Unlike the uniform results produced using the Zipf law, using our “word energy” distribution technique we find variations in the power-law behavior. We also examine some common features that span across languages and identify some intriguing questions concerning how to determine when a text is suitable for its intended readership.

## Introduction

Scaling laws have been an important topic in the physics community across a wide range of fields [1]–[3]. The dynamics of several complex systems in biology [4]–[7], economics [8], [9], and natural phenomena [3], [10] have been described with relative success using scaling laws. Scaling phenomena also emerge in the analysis of data associated with human behavior, especially those containing a statistically distributed component, such as the number of links in the World Wide Web or the size of cities [11], [12]. In current research, the analysis of scaling in data continues to produce new and interesting findings in a variety of scientific fields [13], [14].

In linguistics, Zipf [17] described another typical example of a power law in data on human behavior. He proposed that the distribution of the effort of both speakers and listeners as they attempt to optimize their communication produces a distinctive distribution, the now well-known Zipf Law. Recent research has analyzed how the Zipf scaling of the word frequency distribution changes over the centuries [15], and how this change is affected by both social and natural phenomena [16]. As is the case for many other scaling laws, the Zipf law can also be used in the statistical analysis of huge data sets from other systems [12], [18]–[20], e.g., the distribution of wealth and income in a given population [21] or the distribution of family names [22].

By examining the word frequency in a given corpus of a natural language Zipf found that a word's frequency is inversely proportional to its rank 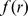 in the frequency table [17], [23], i.e., 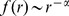, where 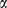 is a constant for the corpus being analyzed. A log-log plot of the frequency distribution for the first 1000–2000 words in the Brown corpus of the English language [24], for example, yields a straight line with slope 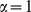 [17]. More recently, Petersen et al. found that the Zipf scaling law for word distribution reveals a significant difference between high-frequency words and low-frequency words, and that this behavior seems to be independent of the language considered [25], i.e., in each regime all languages show the same slope.

In another recent publication, by assuming that the Zipf law is also controlled by the Maxwell-Boltzmann (M-B) distribution associated with the physical world, Miyazima et al. were able to determine a book's linguistic “temperature” value [26], and used this concept to compare the “temperatures” of educational textbooks in the English language. They found that the higher the vocabulary grade level of a textbook, the lower its temperature. They found, for example, that the temperature of English textbooks for grades 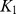 through 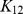 in the US educational system decreases from 1.48 K to 0.87 K when the 1.00 K temperature of the American National Corpus (ANC) is used as a standard. In the same analysis they found that the temperature of Einstein's *The Theory of Relativity* was approximately 0.65 K [26].

If the temperature measurement of a text in a textbook allows us to determine its academic level from its vocabulary, the next step is to determine whether that temperature value can serve as a measurement of the vocabulary complexity of books in general. We propose a technique based on the temperature concept that allows us to analyze texts in their various translations and determine how vocabulary features change across languages. We examine a group of popular books in six different languages and find some intriguing patterns in their translated versions. By improving our comparative analysis, we are able to measure a text's suitability for its intended readership and thus to determine which vocabulary standards better fit a particular text.

## Methods

### Word Energy and Measurement of Temperature

Through the use of some basic concepts, we can define the key quantities. In thermodynamics, the probability function for the energy states in a substance follows the Maxwell-Boltzmann distribution. In general, 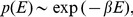
(1)where 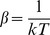, 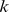 is the Boltzmann constant (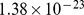 J/K), and 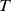 the absolute temperature. Here, as a convenience, we consider 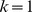 irrespective of unit.

We assume that each word corresponds to an energy value in the M-B distribution in Eq. (1). Although we can only calculate 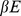 for each word and not 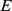 itself, if we assume a 1 K temperature for the corpus considered (e.g., the Brown corpus), we can determine the specific energy for each word.

When we count each word in the vocabulary of a volume of an English text, e.g., a journal, a novel, or a school textbook, we assume that we will find a word distribution that deviates from the distribution of the vocabulary in the Brown English corpus. We use this deviation to determine the temperature of the text in its English version. Fitting our “word-energy” frequency distribution, 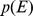 versus 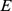, to the Brown corpus, we find a straight line with slope 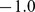 in a semi-log plot, reflecting the standard M-B distribution. Fitting the same “word-energy” distribution to any other text in the same scale, we find a slope sightly higher or lower than the standard. Since this slope represents the term 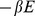 in Eq. 1, we can easily calculate the corresponding energy for this particular text. We fit the distribution to the Maxwell-Boltzmann distribution, change the temperature, and calculate the temperature of the text.

**Figure 1:**
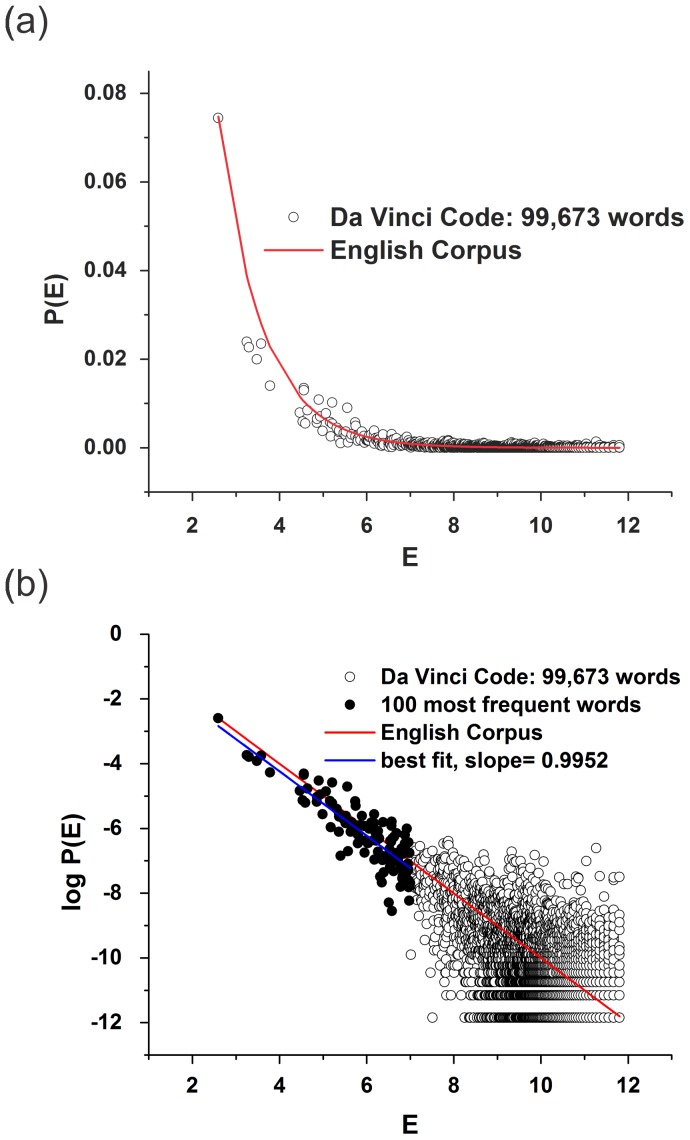
Comparison between distributions of energies for a book and for the corpus standard. The (a) plot of the probability distribution 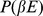 versus the “energy” 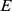 associated to a given word in the vocabulary of the book *The da Vinci Code* by Dan Brown in its English version, compared with the standard curve for the Brown corpus; and (b) its respective semi-log plot. This book contains 99,673 distinct words, named as “items” at the plot that shows only 5,529 different words. To calculate the fit (green line), we considered only the points in red (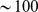), where we choosed the maximum energy as being lower than 7. An increase in the number of points up to 1,000 (interval where the Zipf law is still valid) shall not change the result in a significant way.

Figure 1(a) shows the probability distribution of 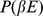 for the vocabulary of the book *The da Vinci Code* by Dan Brown in English where, e.g., 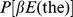, 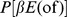 are plotted against the word energies of “the” and “of.” This plot also presents the comparative standard distribution, in this case the energies associated with the words in the Brown corpus. Figure 1(b) shows that it is easier to plot 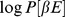 against 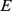 and fit it using a straight line. Note that the “word-energy” distribution for the Brown corpus has the expected slope 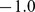, but that the slope for the book is 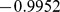, which corresponds to a temperature 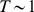. This temperature varies greatly when other books and their translations are considered.

### The Comparative Thermo-Linguistics Technique

The main component of our technique, “comparative thermo-linguistic analysis,” assumes that every readership (e.g., a geographic community or a group of people with common interests) has its own vocabulary. For example, the way in which a newspaper reports an event such as a soccer game is strongly influenced by the frame of reference of its reading public. This goes beyond simply hometown papers supporting the home team. The reading level and interests of those reading the sports page in an up-scale broadsheet will differ from those reading the same in a tabloid, for example.

Our comparative technique for text analysis is as follows:

1. Define the target readership.
2. Determine the standard vocabulary for the target readership, i.e., locate a literary “corpus” that adequately represents its vocabulary. Miyazima et al. [26] considered the corpus of the entire English language as a general standard for the analysis of English textbooks. Their choice was useful, but only in a limited way.
3. Calculate the corresponding “energy” for each word in the corpus in order to determine the standard distribution of word energy for the target readership.
4. Use this energy distribution to determine the “relative temperature” of each text to be examined.
5. Compare the relative temperature of the texts examined with the standard vocabulary exhibited by the literary corpus being used as a reference.

Similar to what we have found for grade levels, we expect the relative temperature of each text to be closely related to the reading effort required of someone in the target readership. When the relative temperature of a text is higher (lower) than that of the standard corpus, the complexity of its vocabulary will be lower (higher) than that of the standard. If the temperatures are approximately the same, the text being examined is deemed highly appropriate for the target readership [see Fig. 1(b)].

## Results and Discussion

### Books and their translations

We next examine how the vocabulary of a text changes when it is translated into another language. To minimize bias, we consider 30 different books and their respective translations (versions) in six different languages. The books include a variety of different authors, release dates, and original languages.

**Figure 2:**
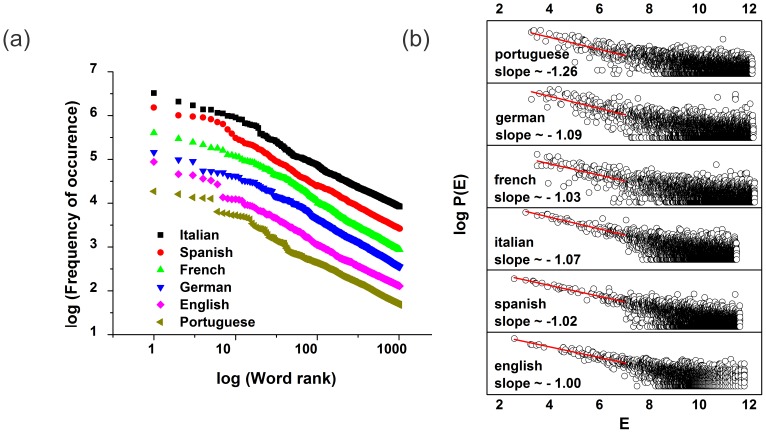
Comparison between the Zipf scaling and the comparative thermo-linguistic technique for several languages. Languages comparision for: (a) a log-log plot exibiting the Zipf law (all curves has similar slopes) in the probability distribution 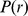 of ocurrency for the 1024 most frequent words in the corpus according the “Project Gutenberg”; (b) and a log plot of the probability distribution 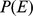 of the word energies in the book *Da Vinci Code* by Dan Brown, exibiting the different slopes, therefore different temperatures. (Note that the y axis in both graphics are shifted for better visualization).

Figure 2a shows a log-log plot similar to Petersen et al. that compares the distribution of the probabilities of occurrence 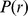 of the 1024 most frequent words indicated in the “Project Gutenberg” corpuses of English, French, German, Portuguese, Spanish, and Italian Languages [25], [27]. Although all of the curves are approximately identical, the rank of a given word (and its corresponding translation) changes when other languages are taken into consideration.

Using our comparative thermo-linguistic analysis we find that the rank position of a word usually differs between languages. Although the Zipf distribution does not change when different languages are considered, when a text is translated the energy distribution does change (see Fig. 2b).

The Figure 2(b) shows a plot of the energy distribution of words for several translated versions of *The da Vinci Code* and their respective temperatures calculated from their slopes (e.g., the slope for the Portuguese translation is approximately 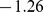, corresponding to 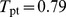, and so on). To allow a comparison between languages, we use 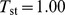 as the “standard temperature” for each corpus.

**Figure 3:**
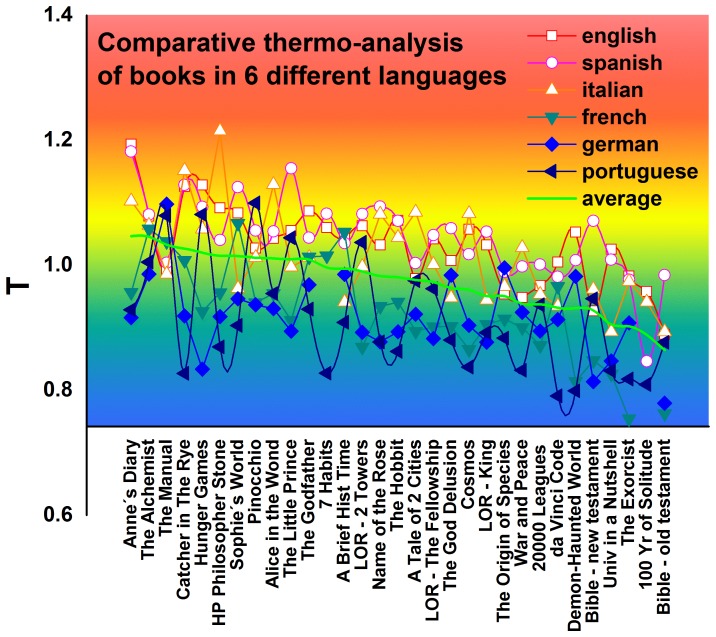
Temperature for books. Plot of characteristic temperature dependance of the language for several books.

We repeat this same procedure for 30 books and their translations into six languages (see Fig. 3). Table 1 shows the numerical results generated by this new technique of “comparative thermo-linguistic analysis.” Figure 3 shows the average temperature, with basic books to the left, medium-level books in the center, and advanced books to the right. Within each of the three regions the arrangement is random.

**Table 1:** Characteristic temperatures for books in several languages.

Book	Author	English	Spanish	Italian	French	German	Portuguese
The Alchemist	Paulo Coelho	1.0784	1.0812	1.0641	1.0582	0.9858	1.0051
The Diary of a Young Girl	Anne Frank	1.1935	1.1819	1.1029	0.9565	0.9161	0.9287
The Manual of the Warrior of Light	Paulo Coelho	0.9926	1.0038	0.9857	1.0380	1.0979	1.0796
The Catcher in The Rye	J. D. Salinger	1.1259	1.1284	1.1517	1.0078	0.9189	0.8270
Pinocchio	Enrico Mazzanti	1.0272	1.0553	1.0124	0.9402	0.9367	1.0996
Hunger Games	Suzanne Collins	1.1282	1.0933	1.0582	0.9259	0.8338	1.0812
Sophies World	Jostein Gaarder	1.0837	1.1250	0.9629	1.0670	0.9464	0.9037
The Godfather	Mario Puzo	1.0868	1.0440	1.0122	1.0134	0.9688	0.9299
Alice in the Wonderland	Charles Lutwidge Dodgson	1.0431	1.0543	1.1293	0.9547	0.9304	0.9547
Harry Potter and the Philosopher Stone	J.K. Rowling	1.0917	1.0405	1.2148	0.9563	0.9180	0.8694
The Little Prince	Antoine de Saint-Exupry	1.0553	1.1549	0.9971	0.9119	0.8947	1.0440
A Brief History of Time	Stephen Hawking	1.0437	1.0354	0.9409	1.0531	0.9855	0.9088
7 Habits of Highly Efficient People	Stephen R. Covey	1.0601	1.0824		1.0152		0.8273
Lord of the Rings - The Two Towers	J. R. R. Tolkien	1.0629	1.0818	0.9979	0.8690	0.8928	1.0366
The Name of the Rose	Humberto Eco	1.0323	1.0939	1.0817	0.9343	0.8776	0.8774
The Hobbit	J. R. R. Tolkien	1.0717	1.0709	1.0444	0.9409	0.8937	0.8623
A Tale of Two Cities	Charles Dickens	0.9785	1.0036	1.0850	0.8945	0.9216	0.9752
Lord of the Rings - The Fellowship of the Ring	J. R. R. Tolkien	1.0418	1.0481	1.0012	0.9017	0.8826	0.9625
The God Delusion	Richard Dawkins	1.0073	1.0589	0.9478	0.9021	0.9842	0.8806
Cosmos	Carl Sagan	1.0575	1.0174	1.0824	0.8652	0.9038	0.8372
The Origin of Species	Charles Darwin	0.9540	0.9895	0.9662	0.9144	0.9957	0.8835
Lord of the Rings - The Return of the King	J. R. R. Tolkien	1.0324	1.0535	0.9441	0.9055	0.8770	0.8922
20000 Leagues Under the Sea	Jules Verne	0.9677	1.0012	0.9536	0.8716	0.8947	0.9366
War and Peace	Leon Tolstoi	0.9482	0.9980	1.0285	0.9004	0.9247	0.8318
The da Vinci Code	Dan Brown	1.0048	0.9815	0.9348	0.9661	0.9131	0.7911
The Holy Bible - New Testament	Several Authors	0.9269	1.0710	0.9616	0.8477	0.8139	0.9464
The Demon-Haunted World	Carl Sagan	1.0530	1.0078		0.8144	0.9824	0.7990
The Universe in a Nutshell	Stephen Hawking	1.0255	1.0090	0.8938	0.8260	0.9254	0.8311
The Exorcist	William peter blatty	0.9827	0.9770	0.9741	0.7543	0.9078	0.8179
One Hundred Years of Solitude	Gabriel Garca Mrquez	0.9579	0.8462	0.9410			0.8093
The Holy Bible - Old Testament	Several Authors	0.8895	0.9844	0.8935	0.7625	0.7795	0.8766

Comparative table of characteristic temperatures values for 31 books in six different languages.

For these results we used frequency lists of up to 10,000 words drawn from a variety of sources and without specific requirements, e.g., how the list was assembled (see Table 2). Figure 3 uses the *Corpus Brasileiro - PUC/SP* [28] as the Portuguese language standard, and the *Brown Corpus* [24] as the English language standard.

**Table 2:** Main features of some corpus in several languages.

Language	Source/Corpus Reference	Number of words	Compilation features
English	Brown Corpus	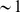 million	500 samples, distributed across 15 genres (mostly novels, and other books).
English	The British National Corpus (BNC)	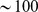 million	Samples of written (90 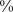 ) and spoken language (10 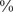 ). from various sources (books, newspaper, dialogues…).
English	Corpus of Contemporary American English (COCA)	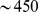 million	Samples equally divided among spoken, fiction, popular magazines, newspapers, and academic texts.
Spanish	Corpus de Referencia del Español Actual (CREA) - Real Academia Española	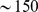 million	Collection of words from books ( 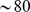 million), and newspapers ( 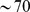 million).
French	Lexique	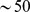 million	Samples of written and spoken language from various sources.
German	Invoke IT	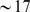 million	Samples of public/free subtitles available at opensubtitles.org.
Italian	Progetto PAISÀ	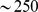 million	Sample texts taken from the web, composed entirely of free texts available.
Portuguese	Corpus Brasileiro - PUC-SP	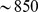 million	Samples of written and spoken language from various sources (books, newspaper, dialogues…).
Portuguese	Corpus de Referência do Português Contemporâneo (CRPC)	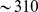 million	Samples from several types of written (literary, newspaper, technical, etc.) and spoken texts.
Portuguese	CETENFolha	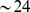 million	Build from electronic texts extracted from the newspaper “Folha de São Paulo”.

Main features comparative table between 10 different corpus in 6 different languages.

**Figure 4:**
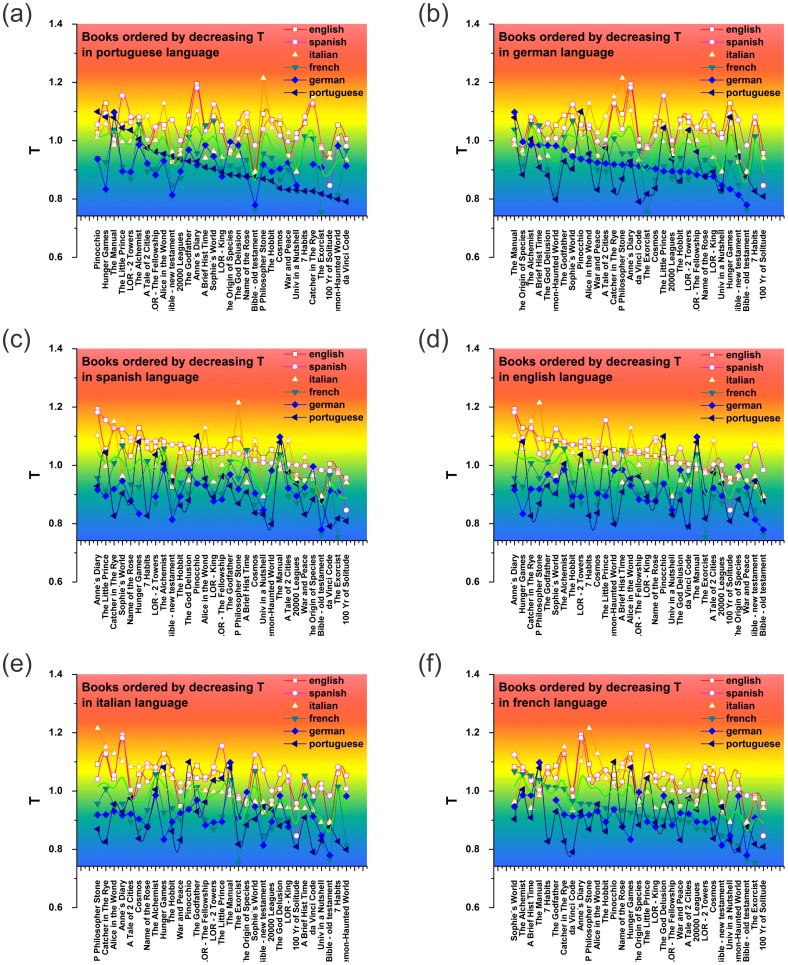
Language dependence of the temperature in the vocabulary of a book. Plots of the characteristic temperature for the vocabulary of books by increasing order in six different languages: (a) Portuguese, (b) German, (c) Spanish, (d) English, (e) Italian and (f) French.

Table 1 shows that, for a given book, the temperature of its Portuguese version is almost always lower than the temperature of the other versions. Exceptions to this include The Bible, The New Testament, and *Pinocchio* (see Fig. 4a). Similar behavior can be observed in the temperature of the German versions of these same books [see Fig. 4(b)]. The English and Spanish language versions, on the other hand, are consistently higher [see Figs. 4(c) and 4(d)]. While the French and Italian versions [see Figs. 4(e) and 4(f)] exhibit temperatures that are intermediate. This result seems to be unaffected by the original language of the book.

### What makes the difference?

Figure 2(b) shows results that imply that the temperature of a book always changes when it is translated. To investigate this we consider books writen by bilingual authors who do their own translations, assuming that the vocabulary preference and literary style of an author will remain constant across translations [29],[30].

Table 3 shows the temperature values for 16 different books, each written and translated into two languages (A and B) by a single bilingual author. Note that in each case the English version of a book tends to have a higher temperature value than the same book in any other language (with only one exception). This result is consistent with the results we obtained when we analyzed the books listed in Table 1, i.e., the translation process *itself* does not significantly affect the change in the complexity of the vocabulary when a book is translated and does not cause the change in temperature.

**Table 3:** Characteristic temperatures for texts written and translated by the own authors.

Book/Text	Author	Language A	Temperature	Language B	Temperature
An Invincible Memory	João Ubaldo Ribeiro	English	1.0306	Portuguese	0.8217
Sergeant Getulio	João Ubaldo Ribeiro	English	1.0874	Portuguese	0.8702
Malone Dies	Samuel Beckett	English	1.1251	French	0.9401
Mercier and Camier	Samuel Beckett	English	1.0545	French	0.8849
Waiting for Godot	Samuel Beckett	English	1.1172	French	0.9286
The Valley	Rolando Hinojosa-Smith	English	0.8508	Spanish	0.9262
Sweet Sweetback's Baadasssss Song	Melvin Van Peebles	English	0.8775	French	0.8455
The Treasure of Sierra Madre	B.Traven	English	1.0269	German	0.8391
The Death Ship	B.Traven	English	1.0202	German	0.8289
Christopher Unborn	Carlos Fuentes	English	0.9241	Spanish	0.9213
The Alchemist	Paulo Coelho	English	1.0784	Portuguese	1.0051
Invisible Cities	Italo Calvino	English	0.9768	Italian	0.8803
Le langage et son double?	Julien Green	English	0.9858	French	0.9051
Instruments of Darkness	Nancy Huston	English	0.965	French	0.8678
Elizabethan Pronunciation	Fausto Cercignani	English	0.9573	Italian	0.9257
La Fourrure de ma tante Rachel?	Raymond Federman	English	0.9607	French	0.8991

Comparative table of characteristic temperatures values for 16 books translated by the own authors.

Examining again the reference corpus of each language, we calculate the temperature of all the books shown in Fig. 3, choosing each corpus irrespective of how it was compiled or assembled. Because the vocabulary of a language is strongly influenced by social and cultural forces [31], a text written for a target readership will be strongly influenced that readership. Thus changes in temperature will occur when there is a change in the standard vocabulary that we use when we do our comparative analysis.

**Figure 5:**
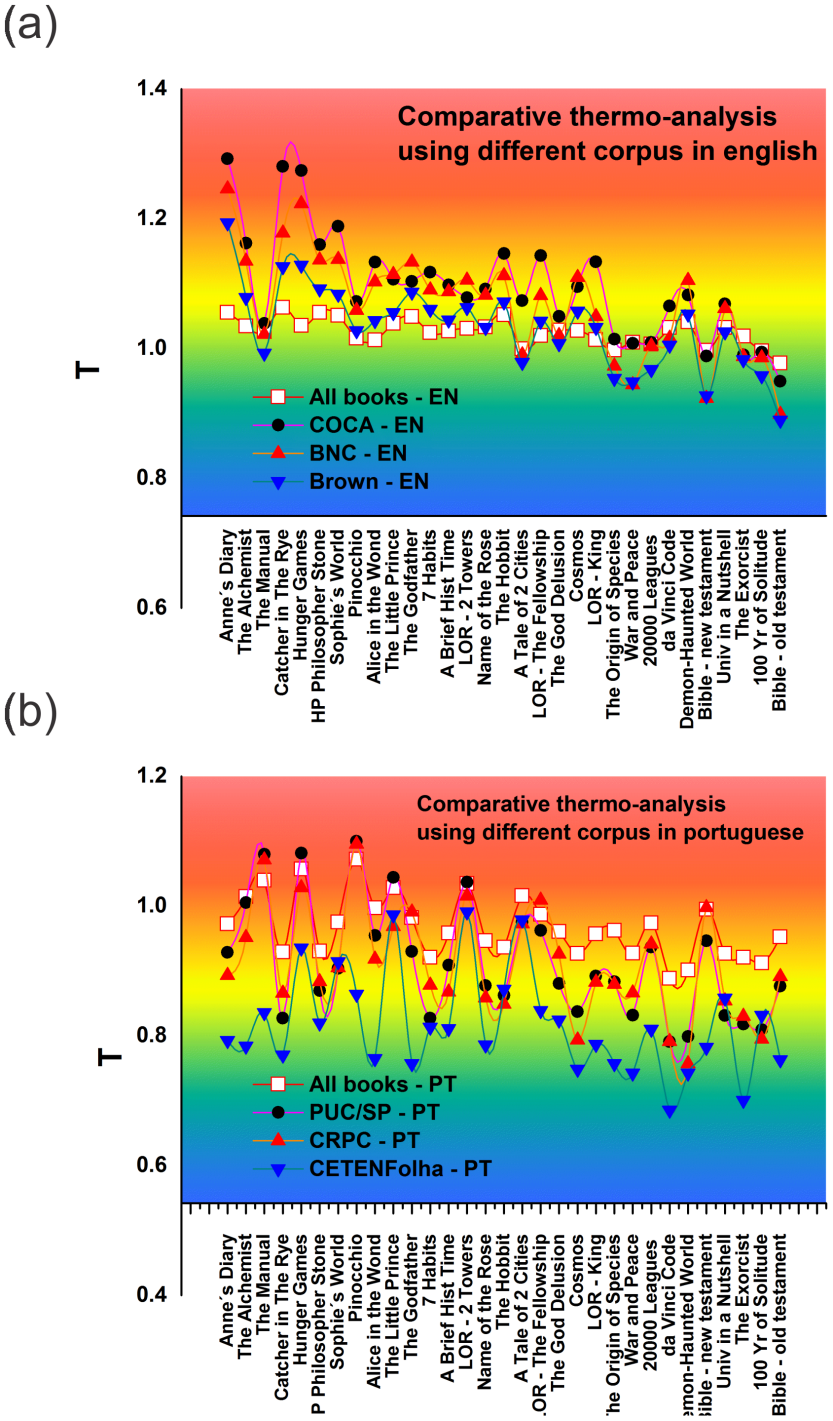
Corpus dependence of the book Temperatures. Plots of the characteristic temperature for books by increasing order for different corpus in (a) English and (b) Portuguese.

Figure 5(a) shows the same set of books shown in Fig. 3 in their English versions. Each curve corresponds to a different corpus. Figure 5(b) shows the Portuguese versions of the same books. We compiled our own corpus using all the words contained in the books in English and Portuguese and used this as our standard corpus in the analysis in both languages–see the curves “All Books EN” and “All Books PT” in Figs. 5(a) and 5(b), respectively.

These figures show that the temperature of the books approaches a value of 1 when we take into consideration our compiled corpus, independent of language, i.e., they are becoming increasingly similar to the baseline corpus. As the vocabulary of a book increasingly deviates from that of the corpus, the temperature deviates from 1.

**Figure 6:**
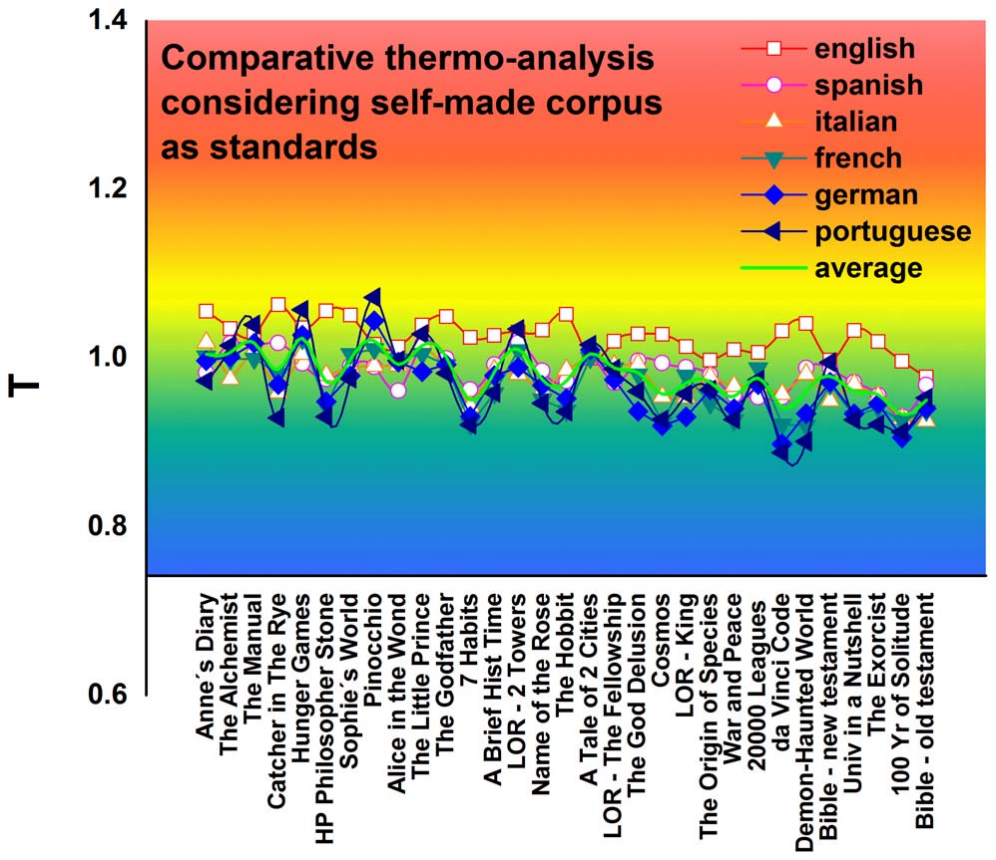
Temperature of books by using self-made corpus. Plots of the characteristic temperature for books considering a self-made corpus with all the books used for each language.

Figure 6 shows a comparison of book temperatures in all six languages using our own compiled corpus. All the temperature values approach 1 and the differences among the languages sharply decrease.

If we were to use a baseline corpus compiled from the words of one book in an analysis of the same book, the temperature would invarably be one. Thus our comparative thermo-analysis technique can be used to measure how appropriate the vocabulary used in a text is to its target readership.

## Conclusions

The temperature of a book is strongly related to its vocabulary level. Basic level textbooks use as many common words as possible and their temperature is higher than the temperature of more advanced books. By performing a cross-language comparisons with our comparative thermo-analysis, we find that this tendency is independent of language, but that the effort required to read or write a given text differs among languages. Figure 3 shows that the temperature of a book in Portuguese is usually lower than the temperature of same text in English. This indicates that the book requires more effort of a Brazilian reader than an English reader.

Figure 6 shows that changing the corpus used as standard will change the effort required of the reader. It also shows that the reading effort never reaches zero, and that books in English always have a higher temperature than books in Portuguese. It is possible that English has a high temperature because there are many synonyms that express a similar content in the English Language, and that Portuguese has a low temperature because it requires more words to express different meanings.

In understand why the temperature of a book, and thus the complexity of its vocabulary, changes when it appears in a different language, we have eliminated the factors of the original language of the author and the influence of the translation process itself. The change that occurs is thus related to the syntax and other grammar features of each language.

Irrespective of cause, a significant factor in solving this puzzle is how well a particular text uses the vocabulary of its target readership. In this way, our comparative thermo-analysis also allows us to determine quantitatively whether the vocabulary of a book, either in its original language or in translation, achieves that goal–how well it reaches its readership.
